# Anatomically specific reactive oxygen species production participates in Marfan syndrome aneurysm formation

**DOI:** 10.1111/jcmm.14587

**Published:** 2019-08-11

**Authors:** Fabian Emrich, Kiril Penov, Mamoru Arakawa, Nathan Dhablania, Grayson Burdon, Albert J. Pedroza, Tiffany K. Koyano, Young M. Kim, Uwe Raaz, Andrew J. Connolly, Cristiana Iosef, Michael P. Fischbein

**Affiliations:** ^1^ Department of Cardiothoracic Surgery Stanford University Stanford California; ^2^ Department of Cardiothoracic Surgery Leipzig University Heart Center Leipzig Germany; ^3^ Department of Cardiovascular Surgery Jichi Medical University Saitama Japan; ^4^ Department of Cardiovascular Medicine Stanford University Stanford California; ^5^ Department of Pathology Stanford University Stanford California

**Keywords:** aneurysm, Marfan syndrome, reactive oxygen species

## Abstract

Marfan syndrome (MFS) is a connective tissue disorder that results in aortic root aneurysm formation. Reactive oxygen species (ROS) seem to play a role in aortic wall remodelling in MFS, although the mechanism remains unknown. MFS *Fbn1^C1039G/+^* mouse root/ascending (AS) and descending (DES) aortic samples were examined using DHE staining, lucigenin‐enhanced chemiluminescence (LGCL), Verhoeff's elastin‐Van Gieson staining (elastin breakdown) and in situ zymography for protease activity. *Fbn1^C1039G/+^* AS‐ or DES‐derived smooth muscle cells (SMC) were treated with anti‐TGF‐β antibody, angiotensin II (AngII), anti‐TGF‐β antibody + AngII, or isotype control. ROS were detected during early aneurysm formation in the *Fbn1^C1039G/+^* AS aorta, but absent in normal‐sized DES aorta. *Fbn1^C1039G/+^* mice treated with the unspecific NADPH oxidase inhibitor, apocynin reduced AS aneurysm formation, with attenuated elastin fragmentation. In situ zymography revealed apocynin treatment decreased protease activity. In vitro SMC studies showed *Fbn1^C1039G/+^*‐derived AS SMC had increased NADPH activity compared to DES‐derived SMC. AS SMC NADPH activity increased with AngII treatment and appeared TGF‐β dependent. In conclusion, ROS play a role in MFS aneurysm development and correspond anatomically with aneurysmal aortic segments. ROS inhibition via apocynin treatment attenuates MFS aneurysm progression. AngII enhances ROS production in MFS AS SMCs and is likely TGF‐β dependent.

## 1 INTRODUCTION

Marfan syndrome (MFS) is a connective tissue disorder (fibrillin‐1 gene mutation) affecting the ocular, musculoskeletal and cardiovascular systems.^1^ Aortic root aneurysm formation and subsequent dissection remain the principal cause of death in MFS patients, with prophylactic surgical replacement (primarily based on size criteria) the only treatment option that effectively increases life expectancy.^2^, ^3^, ^4^ The pathophysiology behind MFS aortic root aneurysm formation is complex and seems related to enhanced transforming growth factor‐β (TGF‐β) signalling.^5^, ^6^, ^7^, ^8^, ^9^ However, the gap in knowledge that explains what happens between TGF‐β signalling and extracellular matrix (ECM) remodelling has prevented the development of innovative treatment strategies.^10^ Further, because TGF‐β stimulates multiple signalling pathways, it is unclear which of these pathways results in aortic aneurysms.^6^, ^11^, ^12^, ^13^, ^14^

Reactive oxygen species (ROS) have emerged as important TGF‐β‐dependent mediators capable of inducing smooth muscle cell (SMC) phenotype switching, SMC apoptosis and matrix metalloproteinase (MMP) activation, all deemed important in ECM remodelling.^15^, ^16^, ^17^, ^18^, ^19^ ROS develop as a by‐product of natural oxygen metabolism and participate in normal cell signalling and homoeostasis.^20^, ^21^ Conversely, elevated ROS levels (oxidative stress) can be harmful and result in detrimental vascular pathologies, including atherosclerosis, hypertension and both abdominal and ascending aortic aneurysms.^22^, ^23^, ^24^, ^25^ Recently, several investigators have reported enhanced ROS within the MFS aortic root aneurysm wall in both human and murine specimens, attributed to increased pro‐oxidant enzymes (NADPH oxidase, nitric oxide synthase and xanthine oxidase) and/or reduced antioxidants (superoxide dismutase).^26^, ^27^ As biological proof that NADPH oxidase‐dependent ROS participate in MFS aneurysm formation, Jimenez‐Altayo et al reported reduced AS aneurysm size and ECM breakdown in *Fbn1^C1039G/^* ^+^/‐Nox4^−/−^ mice.^28^ Importantly, the unanswered question remains why aneurysms localize exclusively to the aortic root in MFS patients despite enhanced TGF‐β throughout the vasculature. This study uniquely reveals enhanced ROS production specific to the aneurysmal aorta, while sparing the normal‐sized descending thoracic aorta in the *Fbn1^C1039G/+^* MFS mouse model. Mechanistically, these anatomic differences are associated with TGF‐β‐dependent NADPH oxidase ROS production both directly and indirectly via angiotensin II (AngII) in MFS ASC‐derived SMC. Finally, direct NADPH oxidase inhibition attenuates MFS aneurysm growth in the *Fbn1^C1039G/+^* MFS mouse model. These data provide important new insights into the mechanisms of early aneurysm formation in MFS and potentially allows for the development of more anatomically directed therapeutics.

## 2 METHODS

### 2.1 Animal studies

Experimental work on animals was performed according to protocols approved by the Administrative Panel on Laboratory Animal Care (APLAC) at Stanford University. Protocols followed the NIH and USDA Guidelines for the Care and Use of Animals in Research. Experiments were performed with *Fbn1^C1039G/+^* mice and C57BL/6J littermate wild‐type controls (WT), equal proportion of male and females, from age 3 to 12 weeks. *Fbn1^C1039G/+^* mice were kindly donated by Dr Harry C. Dietz, Johns Hopkins University School of Medicine.

### 2.2 Echocardiography

Transthoracic echocardiography (TTE) was performed at age 3 weeks (baseline) and then weekly until age 12 weeks (n = 7‐8). Mice were sedated with 2% inhaled isoflurane (Baxter Healthcare Corporation) delivered via nose cone. The aorta was imaged in the parasternal long‐axis view using a Vevo 770 Imaging Station (VisualSonics Inc) equipped with a Vevo 2100 system and the ultra‐high frequency liner array transducer RMV 704. For statistical analysis, measurement of the aortic diameter (from edge to edge, at the largest portion of the AS aorta) was performed using three technical replicates for each animal by two blinded investigators.

### 2.3 Apocynin (Acetovanillone) treatment, in vivo

*Fbn1^C1039G/+^* and littermate WT mice were treated with (a) oral apocynin (Acetovanillone, 4‐hydroxy‐3‐methoxyacetophenone, 98%, Acros Organics, NJ, USA) (0.4 mg/mL) dissolved in drinking water or (b) vehicle control beginning at 3 weeks until 12 weeks when the animals were killed. Some animals monitored by echo over time died before completing 12 weeks of treatment.

### 2.4 Aortic tissue preparation

Mice were killed by inhalation of an overdose of isoflurane delivered via nose cone. The aorta was dissected and divided into the following segments: (a) aortic root (distal to aortic valve), ascending and arch (AS) and (b) descending thoracic aorta (DES). Samples were used for multiple purposes: (a) to determine enzymatic activity in fresh tissues (chemiluminescence reading), (b) cryopreservation for later investigation, (c) smooth muscle cell isolation (by enzymatic digestion), (d) histologic analysis (dihydroethidium [DHE] staining), (e) in situ zymography and (f) Verhoeff's elastin‐Van Gieson [EVG] staining of fresh tissues.

### 2.5 Fbn1^C1039G/+^‐derived SMC

Aortic SMCs were isolated from 4‐week‐old *Fbn1^C1039G/+^* and littermate WT control mice, cultured in SMC media (SmGM‐2 Bullet Kit CC3182; Lonza) and kept at 37°C with 5% CO_2_. Prior to treatment, SMC purity was confirmed by flow cytometry analysis of SMC‐specific markers. All experiments were conducted on SMC at passages 3‐6. SMC were depleted of growth factors for 24 hours and kept in medium supplemented with 0.2% foetal bovine serum for 24 hours, then cultured for another 24 hours in testing conditions.

### 2.6 In vitro detection of ROS in SMCs treated with TGF‐β neutralizing antibody treatment, in vitro

For ROS detection, a lucigenin chemiluminescence test was used on SMC for 24 hours in medium supplemented with either (a) anti‐TGF‐β (1, 2, 3) antibody (20 µL/mL) (msMab IgG1 Clone #1D11, MAB1835, R&D Systems); (b) angiotensin II (AngII) (1 µmol/L/mL) (Sigma‐Aldrich); (c) anti‐TGF‐β antibody + AngII; or (d) isotype control IgG antibody.

### 2.7 EVG staining

The aorta was dissected and fixed in 4% paraformaldehyde (PFA). Isolated tissue was embedded in Tissue‐Tek OCT Compound Histomount, and serial cryosections of 5μm thickness were displayed in groups of three on the same histological slide. The slides were stained with Accustain EVG kit (Sigma‐Aldrich, St. Louis, MO, USA) and imaged at 10x and 40x magnification using a Leica DM4000B microscope.^12^ The elastic lamina in three AS and DES aortic sections per slide were assessed by a blinded pathologist for the following: (a) average number of breaks per elastic lamina by counting them circumferentially in all lamina; and (b) semi‐quantitative assessment of elastic lamina thinning on a scale of: 0 = none, 1 = mild; 2 = moderate; 3 = severe (ie to the point of multi‐perforate in areas).^12^ Experiments included n = 9 mice for control and n = 10 for apocynin‐treated groups, and have been done in triplicate using three consecutive sections of the AS and the DES aorta from each animal (a total of 3 × 3 = 9 tissue sections/animal). Animals allocated for histological evaluation were different from mice assigned for echo measurements. For statistical analysis, each data set consists of nine biological replicates for control and 10 biological replicates for apocynin and nine technical replicates (3 × 3 tissues per mouse) analysed by blinded investigators.

### 2.8 Superoxide identification by dihydroethidium (DHE) staining

Freshly dissected mouse aortic segments were embedded in Tissue‐Tek OCT Compound Histomount and frozen on dry ice. The aortic segments were sectioned (7 μm). A single sample was incubated with superoxide dismutase‐polyethylene glycol (SOD‐PEG) (Sigma‐Aldrich) and stained with DHE (50 μL per sample) (AnaSpec Inc)^29^ to confirm no autofluorescence. One sample per slide was left unstained as negative control for the staining procedure. Samples were placed in a dark humidity chamber at 37°C for 30 minutes. After washing with PBS 1×, a solution of 70% glycerol was used to mount a glass coverslip onto the tissues. Samples were kept at 4°C until imaging. Specimens were imaged at 10× and 40× magnification using a Leica DM4000B fluorescent microscope at excitation and emission wavelengths of 520 and 610 nm, respectively. ImageJ software (NIH open source) was used to outline cells manually in the region of interest (ROI) and quantify integrated pixel intensity (mean intensity/area). Reciprocal ROI from unstained samples was used to subtract the background noise. Triplicate experiments included n = 6 mice per group, using three consecutive sections of the AS and the DES aorta from each animal. Statistical analysis has been done on data sets including five biological replicates (n = 6 mice) and nine technical replicates (3 × 3 tissue sections).

### 2.9 Evaluation of ROS production in mouse aortic tissue using lucigenin chemiluminescence assay

Mice were killed, and the aorta was dissected and prepared, as noted above. All specimens were kept in cold PBS solution (PBS pH 7.4, Gibco, Life Technologies Corporation) until use. Superoxide production in whole mouse aortic tissue was measured by lucigenin‐enhanced chemiluminescence using a single‐tube luminometer (Berthold FB12, Titertek‐Berthold, Berthold Detection Systems GmbH) modified to maintain the sample temperature at 37°C. Basal chemiluminescence from whole AS and DES mouse aortic tissue (n = 6 per group) was measured in PBS buffer (2 mL) containing 4 μL lucigenin (5 µmol/L) after reaching equilibrium (7 minutes). After application of 20 µL NADPH (100 µmol/L), the chemiluminescence signal in each AS and DES aorta reached a plateau within 5 minutes. Therefore, the measurements in this preparation do not reflect the maximal NADPH‐stimulated superoxide production.^30^

### 2.10 Measurement of ROS production in murine vascular SMC using lucigenin chemiluminescence assay

Superoxide production in mouse vascular SMC isolated from the AS and DES aortas, respectively, was measured by lucigenin‐enhanced chemiluminescence in a 96‐well plate system and multi‐mode microplate BioTek Synergy H1 Hybrid Reader (BioTek Instruments Inc) at 37°C. Investigators have reported that the lucigenin chemiluminescence assay signal is not generated by NADPH oxidase activity alone, but also via the cytochrome system (add reference). SMC were cultivated in a 6‐well plate until they reached 90%‐95% confluence, then starved 24 hours in medium with 0.2% FBS and finally submitted to different treatments for another 24 hours.^30^ The SMC monolayer was disrupted with Gibco TrypLE^TM^ (Thermo Fisher Scientific), and SMCs were automatically counted (Invitrogen Countess™ automated cell counter, Life Technology Corporation). Cells were resuspended (100 000 cells/well) in a 96‐well plate in warm (37°C) PBS 1× (480 µL). Basal chemiluminescence signal from SMCs (AS and DES) was evaluated by using Gen5 Version 2.03 software (BioTek Instruments Inc) in PBS buffer (160 µL) at one time‐point (0 seconds). After adding 40 µL of lucigenin (5 µmol/L), measurements were taken at four time‐points (0, 30, 60 and 90 seconds). Finally, 20 µL of NADPH (100 µmol/L) was added and serial measurements were recorded at 13 consecutive time‐points every 30 seconds (from 0 to 540 seconds).^30^ Light detection was possible after the first 30 seconds, and the signal was stabilized after 60‐300 seconds. Time‐point measurements between 90 and 300 seconds were used for statistical analysis.

### 2.11 In situ zymography

Protease activity was measured in fresh frozen aortic tissue using Dye Quenched Gelatin (DQ‐Gelatin) (Invitrogen). Freshly harvested aortic samples were embedded in Tissue‐Tek OCT Compound Histomount without fixation and cut into 5 µm thick serial sections. A photographic emulsion‐based approach for in situ zymography (ISZ) was used. Low‐melting agarose (1%) (Sigma‐Aldrich) was dissolved in PBS (Gibco Life Sciences). DQ‐Gelatin (1 mg/mL) was diluted 1:10 in the low‐melting agarose solution. Agarose solution with or without DQ‐Gelatin (200 mL) was then placed on the aortic tissue sections and covered with a 22 x 40 mm glass coverslip. Sections were placed at 4°C for 30 minutes to solidify the agarose and then incubated at 37°C for 24 hours. Specimens were visualized by conventional microscopy, and images were captured at an exposure time of 50 msec.^31^ ImageJ software (NIH open source) was used to outline cells manually in the ROI and quantify integrated pixel intensity (mean intensity/area). Protease activity was measured by calculating the difference in light emission from the ROI between DQ‐Gelatin‐containing sections and autofluorescence in sections without DQ‐Gelatin (n = 5 per group). Data resulted from the examination of three technical replicates statistically averages and integrated for graphic plots.

### 2.12 Statistical analysis

Statistical analysis was performed using StatPlus:mac Pro (AnalystSoft Inc for Mac OS^®^. version v6). All data throughout the study are presented as mean ± standard deviation (SD). Results are compared to age‐matched, littermate WT controls or vehicle control‐treated subjects, if not otherwise stated. Non‐parametric tests were used for comparisons. Mann‐Whitney U test was used for comparison of two groups for independent samples (*Fbn1^C1039G/+^* versus WT, and treatment versus control). Wilcoxon signed‐rank test was used for comparison of two dependent samples (ASC versus DESC from the same animal). Only *P* < .05 values were considered significant regardless of the two sources of variation.

## 3 RESULTS

### 3.1 Enhanced ROS is specific to the AS aorta in *Fbn1^C1039G/+^* MFS mice

**Figure 1:**
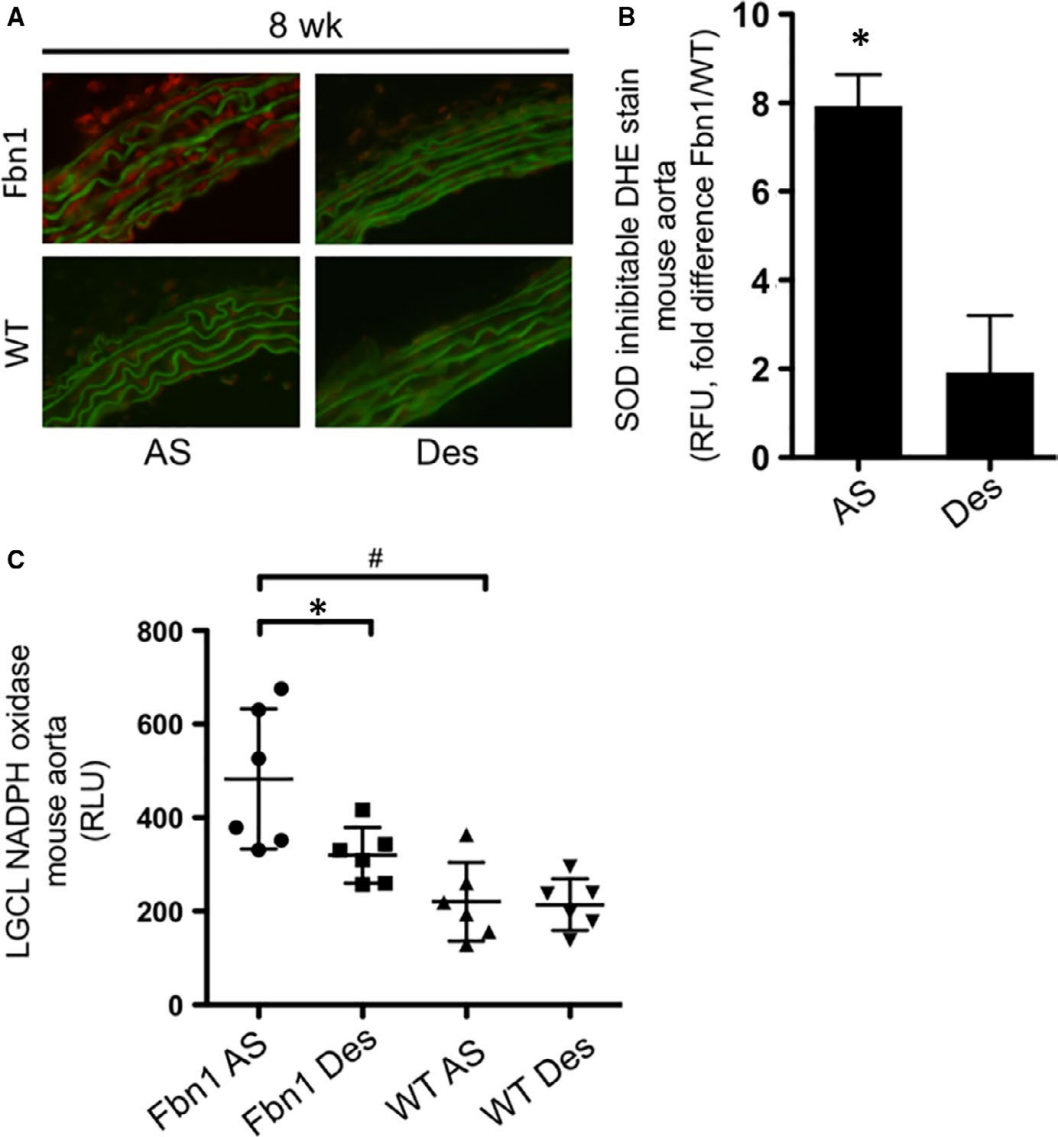
A, Representative image of DHE staining (red) in 8‐wk‐old *Fbn1^C1039G/+^* (Fbn1) and wild‐type donor control (WT) mouse aortic root/ascending (AS) and descending (Des) aortas. B, Quantification of DHE staining in Fbn1 and WT mouse AS and Des aorta (n = 6, each group, *P = .02), expressed as RLU fold difference (Fbn1/WT). C, Quantification of NADPH oxidase activity by LGCL in tissue from 8‐wk‐old Fbn1 and WT mouse AS and Des aorta (n = 6, each group). RLU = relative light units. (*P = .046, ^#^ *P* = .016)

Recapitulating the pathology seen in human MFS aortic root aneurysms, *Fbn1^C1039G/+^* mice reproducibly develop AS aortic aneurysms. To study early aneurysm formation, eight‐week‐old *Fbn1^C1039G/+^* mice were examined for ROS activity. To determine whether enhanced ROS production was anatomically specific to the *Fbn1^C1039G/+^* aneurysmal aorta, we compared DHE staining in the AS and Des aortas from both *Fbn1^C1039G/+^* and littermate WT control mice (Figure 1A). ROS was significantly increased in *Fbn1^C1039G/+^* AS aorta compared to *Fbn1^C1039G/+^* normal‐sized Des aorta, as well as WT AS and Des aortic specimens (*Fbn1^C1039G/+^* AS: 7.93 ± 0.71‐fold versus WT AS, n = 6, *P* = .02; *Fbn1^C1039G/+^* Des: 1.91 ± 1.28‐fold versus WT Des, n = 6 each group, *P* = .12) (Figure 1B). Similarly, LGCL revealed increased NADPH activity in the *Fbn1^C1039G/+^* AS aorta only (*Fbn1^C1039G/+^* AS: 482.43 ± 195.87 RLU; *Fbn1^C1039G/+^* Des: 300.32 ± 113.73 RLU; WT AS: 230.27 ± 44.89 RLU; WT Des: 226.74 ± 55.03 RLU, n = 6 each group, *P* = .046 for *Fbn1^C1039G/+^* AS versus WT AS and *P* = .016 for *Fbn1^C1039G/+^* AS versus *Fbn1^C1039G/+^* Des) (Figure 1C).

### 3.2 Treatment with the inhibitor apocynin reduces AS aneurysm formation in the *Fbn1^C1039G/+^* mouse model

**Figure 2:**
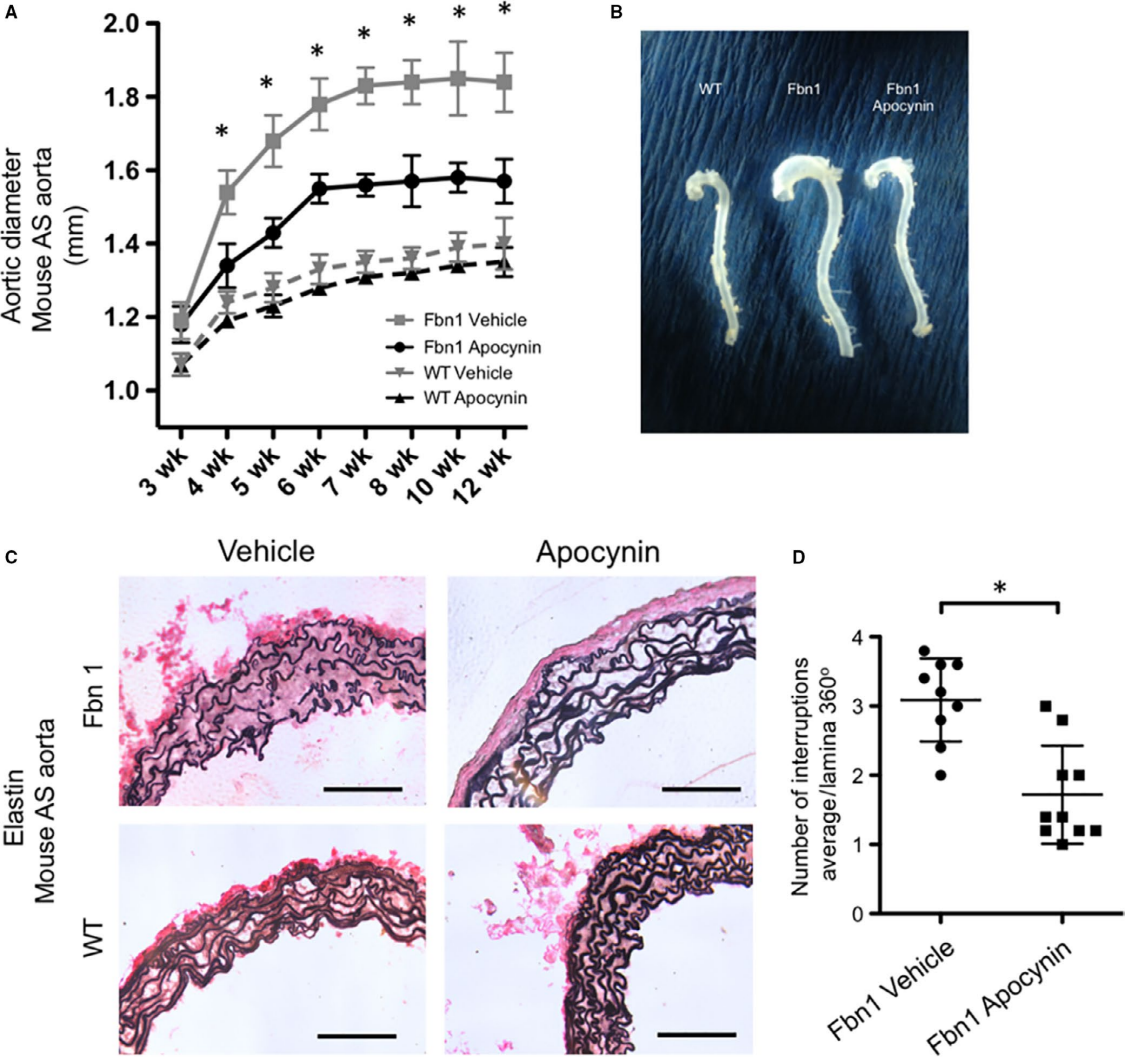
A, Echo measurements of Fbn1 and WT mouse AS aorta comparing apocynin treatment versus untreated control (n = 7 for apocynin treated; n = 8 for untreated *Fbn1^C1039G/+^* mice, *P < .03). B, Representative image of explanted aortas from 12‐wk‐old mice (WT, untreated Fbn1 (vehicle) and apocynin‐treated Fbn1 mice. C, Representative Verhoeff's elastin‐van Gieson (EVG) stain of AS aortas at 8 wk comparing untreated *Fbn1^C1039G/+^* and WT (vehicle) versus apocynin‐treated *Fbn1^C1039G/+^* and WT mice (apocynin). D, Average number of interruptions per elastin lamina in the entire AS aortic circumference from untreated *Fbn1^C1039G/+^* (vehicle) versus apocynin‐treated *Fbn1^C1039G/+^* mice (apocynin) (n = 9 for control and n = 10 for apocynin, * *P* = 0.002) at age 8 wk

As biological proof of concept that ROS contribute to aneurysm formation in MFS mice, *Fbn1^C1039G/+^* and WT control mice were treated with the unspecific NADPH oxidase inhibitor apocynin from ages 3 to 12 weeks. Apocynin significantly reduced aneurysm formation in *Fbn1^C1039G/+^* mice by 4 weeks when compared to untreated *Fbn1^C1039G/+^*, although the diameters do not return to WT control. Apocynin did not alter aortic growth in WT control mice (WT values not shown) (*Fbn1^C1039G/+^* apocynin versus *Fbn1^C1039G/+^*untreated: 1.34 ± 0.15 versus 1.54 ± 0.17, *P* = .03 at 4 weeks; 1.43 ± 0.1 vs 1.68 ± 0.2, *P* = .008 at 5 weeks; 1.55 ± 0.1 vs 1.78 ± 0.19, *P* = .013 at 6 weeks; 1.56 ± 0.09 vs 1.83 ± 0.14, *P* = .002 at 7 weeks; 1.57 ± 0.19 vs 1.84 ± 0.17, *P* = .015 at 8 weeks; 1.58 ± 0.12 vs 1.85 ± 0.27, *P* = .032 at 10 weeks; and 1.57 ± 0.05 versus 1.84 ± 0.24, *P* = .015 at 12 weeks, n = 7 for apocynin treated and n = 8 for untreated *Fbn1^C1039G/+^* mice) (Figure 2A,B). Histological characterization with EVG staining of AS aortic specimens revealed severe elastin fragmentation in *Fbn1^C1039G/+^* mice at 8 weeks (3.09 ± 0.6 breaks/lamina, n = 9). Correlating with reduced aneurysm size, apocynin significantly reduced elastin breakdown in *Fbn1^C1039G/+^* mice (1.72 ± 0.71 breaks/lamina, n = 10, *P* = .002) (Figure 2C,D). The increase in elastin fragmentation evident on histological sections suggests that heightened proteolytic activity may contribute to aneurysm formation in *Fbn1^C1039G/+^* mice.

**Figure 3:**
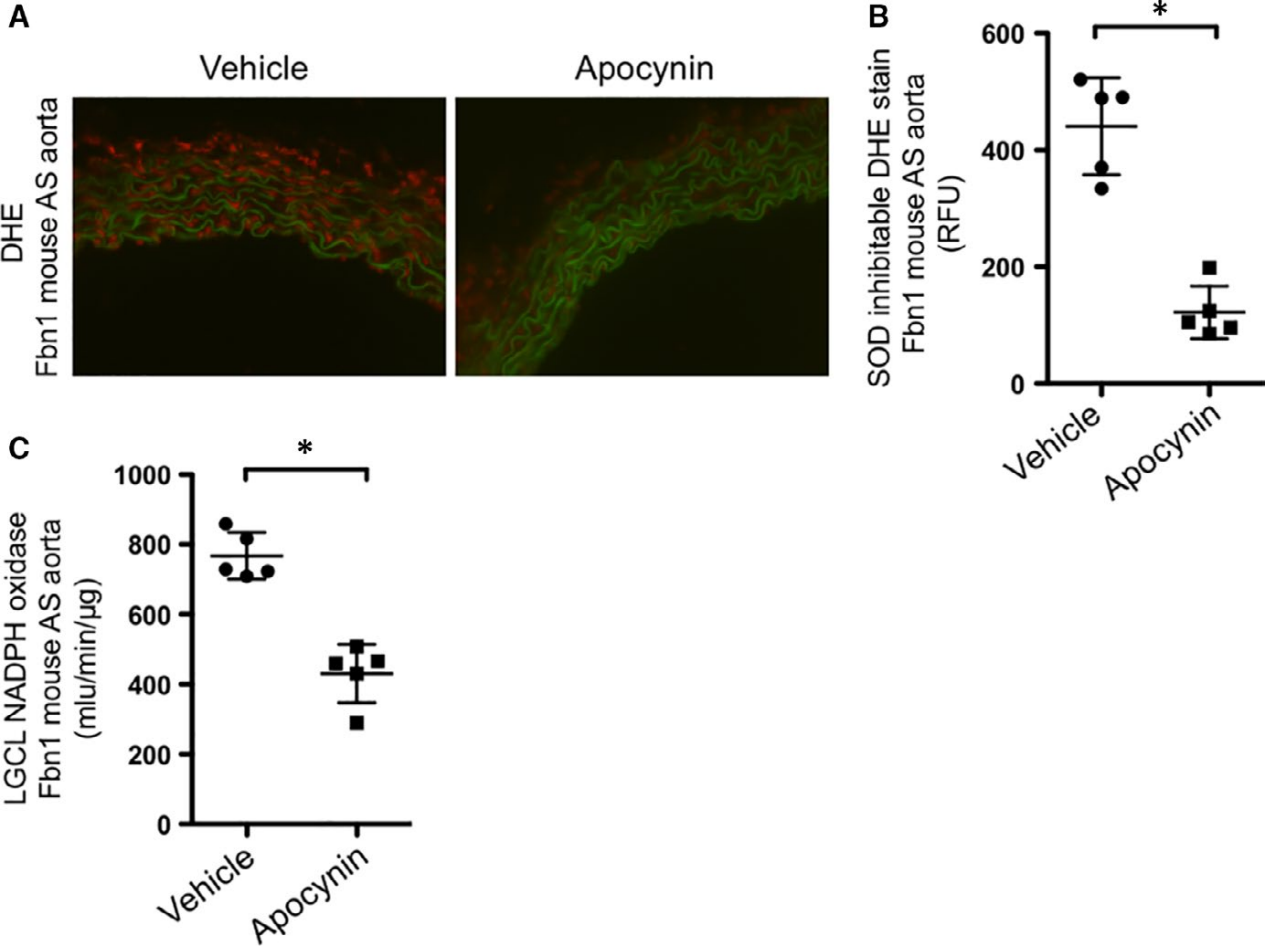
A, Representative image of DHE staining (red) in 8‐wk‐old Fbn1 mouse AS aorta treated with vehicle vs apocynin. B, Quantification of DHE staining in 8‐wk‐old Fbn1 mouse AS aorta treated with vehicle vs apocynin (n = 5, each treatment group, *P = .02). C, Quantification of NADPH oxidase activity by lucigenin chemiluminescence (LGCL) in tissue from 8‐wk‐old Fbn1 mouse AS aorta treated with vehicle vs apocynin (n = 5, each treatment group, *P = .001)

**Figure 4:**
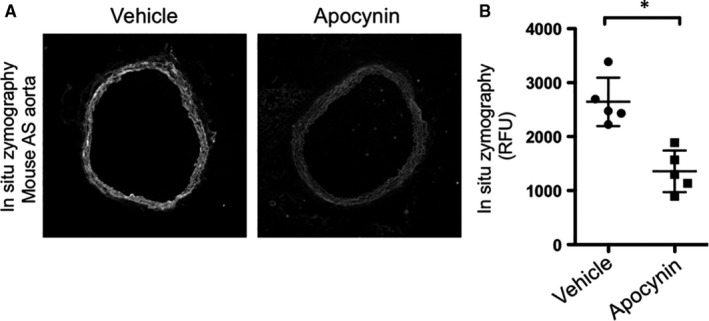
A, Representative image of in situ zymography in 8‐wk‐old *Fbn1^C1039G/+^* mouse AS aorta treated with vehicle vs apocynin. B, Quantification (RFU) of in situ zymography in 8‐wk‐old *Fbn1^C1039G/+^* mouse AS aorta treated with vehicle vs apocynin (n = 5, per treatment group, *P = .02)

To confirm that NADPH oxidase inhibition reduced ROS production, both DHE and LGCL were tested in apocynin‐treated and untreated *Fbn1^C1039G/+^* mice. As hypothesized, apocynin significantly decreased NADPH oxidase activity and ROS production (LGCL *Fbn1^C1039G/+^* apocynin: 430.72 ± 66.41 RLU; untreated *Fbn1^C1039G/+^*: 766.94 ± 83.49 RLU, n = 5 each group, *P* = .001; DHE *Fbn1^C1039G/+^* apocynin: 121.68 ± 44.99 RLU; untreated *Fbn1^C1039G/+^* 440.69 ± 82.95 RLU, n = 5 each group, *P* = .02) (Figure 3A,B,C). Finally, to test our hypothesis that ROS increases protease activity, in situ zymography was performed on apocynin‐treated and untreated *Fbn1^C1039G/+^* AS aortic specimens. Apocynin treatment significantly reduced protease activity within the *Fbn1^C1039G/+^*aortic wall compared to untreated *Fbn1^C1039G/+^* mice (*Fbn1^C1039G/+^* apocynin: 1356.17 ± 398.73 RFU; untreated *Fbn1^C1039G/+^*: 2665.78 ± 458.99 RFU, n = 5, each group, *P* = .02) (Figure 4A,B).

### 3.3 AngII increases NADPH oxidase activity in aortic AS SMC, in vitro

**Figure 5:**
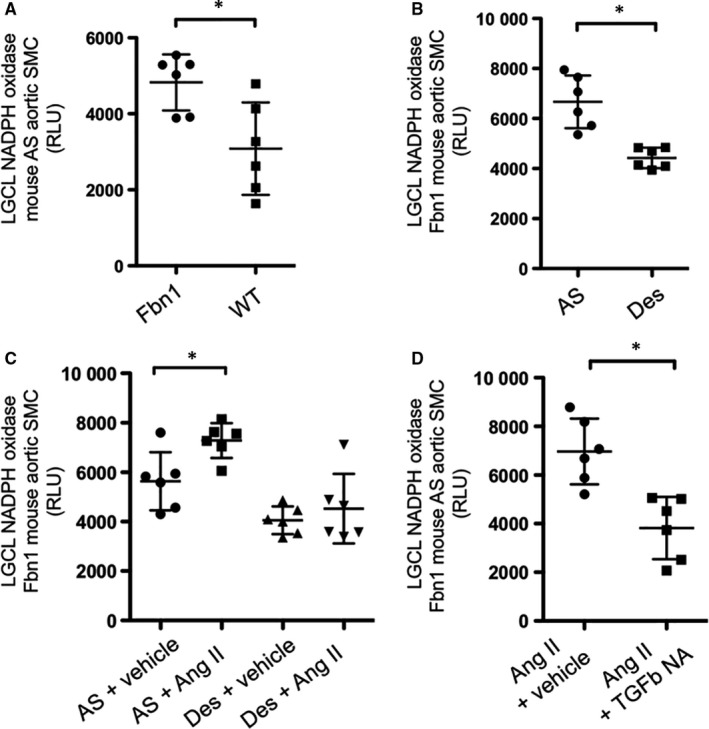
A, In vitro quantification of NADPH oxidase activity by lucigenin chemiluminescence (LGCL) in *Fbn1^C1039G/+^* (Fbn1) aortic AS and Des‐derived smooth muscle cells (SMC) (n = 6, *P = .02). B, In vitro quantification of NADPH oxidase activity by LGCL in Fbn1 aortic AS and WT AS SMC (n = 6, *P = .03). C, In vitro quantification of NADPH oxidase activity by LGCL in Fbn1 AS and Des SMCs treated with AngII or vehicle (n = 6, *P = .03). D, In vitro quantification of NADPH oxidase activity by LGCL in Fbn1 AS SMCs treated with AngII and with either TGF‐β neutralizing antibody (TGFb NA) or vehicle (n = 6, *P = .009)

Previous investigators reported that enhanced TGF‐β signalling causes aneurysm formation in MFS mice, and reduced following losartan treatment (AngII receptor blockade) (5‐14). To define a mechanistic link between TGF‐β signalling, AngII and NADPH oxidase activity in the MFS mouse model, we performed in vitro studies using SMCs derived from either *Fbn1^C1039G/+^* (a) AS or (b) Des aortas. Confirming our in vivo studies, LGCL showed a significant increase in NADPH activity in *Fbn1^C1039G/+^* ^^AS compared to Des SMCs (*Fbn1^C1039G/+^* AS SMC: 6667.25 ± 1317.62 RFU; *Fbn1^C1039G/+^* Des SMC: 4442.58 ± 377.78 RFU, n = 6, *P* = .02) (Figure 5A). The increased NADPH activity is specific to *Fbn1^C1039G/+^* ^^AS SMC, but not WT AS SMC (*Fbn1^C1039G/+^* AS SMC: 4825.50 ± 736.55 RFU; WT AS SMC: 3084.33 ± 1214.64 RFU, n = 6, *P* = .03) (Figure 5B). To investigate the impact of AngII on NADPH activity, we treated *Fbn1^C1039G/+^* ^^SMCs with AngII for 24 hours and then performed LGCL. NADPH activity was significantly increased following AngII treatment in *Fbn1^C1039G/+^* ^^AS, but not Des SMCs (*Fbn1^C1039G/+^* AS SMC + AngII: 7286.50 ± 703.26 RFU; *Fbn1^C1039G/+^* DES SMC + AngII: 4531.17 ± 1407.85 RFU, n = 6, *P* = .03) (Figure 5C). NADPH activity was reduced in AngII‐treated *Fbn1^C1039G/+^* ^^AS SMCs after TGF‐β neutralizing antibody (Nab) (*Fbn1^C1039G/+^* AS SMC + AngII: 6972.59 ± 1352.13 RFU; *Fbn1^C1039G/+^* AS SMC + AngII+TGF‐βNAb: 3820.42 ± 1284.65 RFU, n = 6, *P* = .009) (Figure 5D). This reduction was AS specific, with no significant decrease noted in DES SMC (*Fbn1^C1039G/+^* AS SMC + AngII+TGF‐β NAb: 4693.08 ± 1585.85 RFU; *Fbn1^C1039G/+^* DES SMC + AngII+TGF‐β NAb: 5778.66 ± 1229.79 RFU, n = 6, *P* = .47).

## 4 DISCUSSION

The specific role TGF‐β plays during aneurysm formation in MFS murine models remains complicated, although most animal studies support a pathologic function through aortic wall remodelling in two different Marfan mouse models (*Fbn1^mgR/mgR^* and *Fbn1^C1039G/+^*).^5^, ^6^, ^7^, ^8^, ^9^, ^10^, ^11^, ^12^, ^13^, ^14^, ^32^, ^33^ Identifying key components within the molecular pathway(s) that lead to aneurysm formation may translate into innovative medical therapies directed at preventing or slowing aneurysm growth 33, 34. Herein, our laboratory reports that ROS contribute to aneurysm formation in MFS. Under normal physiological conditions, ROS play an important role as signalling molecules in endothelial function and vascular tone.^35^, ^36^, ^37^ In contrast, excessive ROS production (oxidative stress) can induce a pathologic environment within the aortic wall, including endothelial dysfunction, inflammation and ECM remodelling.^21^, ^38^ The novel findings in this study are as follows: (a) ROS are elevated in MFS aortic aneurysms in the *Fbn1^C1039G/+^* ^^mouse model; (b) ROS enhancement parallels anatomically with aneurysmal aortic segments, specifically; (c) ROS reduction via apocynin reduces aneurysm formation in *Fbn1^C1039G/+^* mice; and (d) SMC ROS production is enhanced via AngII and likely TGF‐β dependent, in vitro.

In this work, we identified enhanced ROS in murine aortic aneurysm samples. As biological proof of concept that ROS contribute to MFS aneurysm formation, *Fbn1^C1039G/+^* mice treated with the unspecific NADPH oxidase inhibitor apocynin exhibited reduced ROS production, which directly correlated with decreased protease activity/ECM destruction and diminished aneurysm formation. Needless to say, it remains possible that apocynin has other beneficial off‐target affects, including acting as a ROS‐scavenger, blocking other elastases or increasing SMC proliferation.^39^ Several investigators have studied the involvement of ROS in thoracic aortic root/ascending aortic aneurysm formation, including individuals with MFS,^24^, ^27^, ^40^, ^41^, ^42^ as well as patients with bicuspid aortic valves.^43^, ^44^, ^45^ The hypothesized important role of ROS in MFS aneurysm formation presented here agrees with the findings of Jimenez‐Altayo et al who also reported increased nitrotyrosine residues in both aortic aneurysms and cultured SMC from MFS patients.^28^ This group identified several possible redox stress mechanistic targets, including alpha‐smooth muscle actin (α‐SMA), encoded by the ACTA2 gene and the cytoskeleton protein, annexin A2. Intriguingly, although we know that patients with ACTA2 genetic mutations can develop familial thoracic aortic aneurysms, this finding suggests that redox modifications to the normal α‐SMA protein may potentially promote aneurysm formation.^28^ ROS can affect multiple additional downstream effectors during AS aneurysm formation, including enhanced (a) MMP activation; (b) SMC apoptosis; and (c) SMC phenotype switching.^42^, ^46^ Here, we describe the important relationship between ROS and protease activity, both in vitro and in vivo. Our laboratory previously presented the contribution of SMC apoptosis to MFS aneurysm formation, although not directly tested here.^11^, ^12^ Of note, because aneurysms still develop despite ROS reduction, other factors must contribute to the pathophysiology triggering MFS aneurysm development.

One of the key findings in this study is that ROS are enhanced in the *Fbn1^C1039G/+^* aneurysmal AS aorta, yet remain at baseline levels in the normal‐sized descending aorta. Importantly, this result rules out the theory that aortic root SMCs merely react distinctively to systemically enhanced ROS compared to the rest of the aorta. Lineage studies have shown that vascular SMC in the different aortic anatomic segments have distinct embryologic origins.^47^ Recent reviews have suggested that the diversity of SMC origin may explain site‐specific location of various vascular diseases, including aneurysm formation.^48^, ^49^ Of note, an important limitation of our animal model is that aneurysms involve the aortic root and ascending aorta in *Fbn1^C1039G/+^* mice, in contrast to the aortic root only in humans. Therefore, when studying the role aortic SMC embryologic lineage plays during aneurysm development in the mouse model, we compare aneurysm to non‐dilated aortic segments. Our laboratory's overarching hypothesis is that *Fbn1^C1039G/+^* AS aorta SMC (second heart field and neural crest) must respond differently to systemically enhanced TGF‐β versus the remainder of the aorta (paraxial mesoderm). More specifically, either superoxide‐generating enzymes are enhanced or free radical scavengers (superoxide dismutase) are reduced in the specific embryologic aortic segments where aneurysms develop. Although the MFS mouse model allows us to study early aneurysm development, an obvious weakness is this study remains that we did not confirm presence of ROS prior to aneurysm formation. Lastly, although not employed in this study, an alternative strategy to test our overarching “aortic embryologic origin” hypothesis includes differentiating induced‐pluripotent stem cells (iPSc) into SMC from each embryologic derivation to study how SMC origin influences ROS production following TGF‐β stimulation.

There are several hypothesized triggers for increased ROS production in the MFS aorta, including enhanced (a) TGF‐β signalling, (b) aortic root stress and strain (increased biaxial loading unique to the ascending aorta) (add Bellini reference) and (c) AngII signalling. AngII, the major peptide of the renin‐angiotensin system, is a potent vasoconstrictor, but also stimulates ROS production via NADPH oxidase (NOX).^50^ Through a positive feedback loop, ROS can increase ATR1 expression, further increasing oxidative stress.^51^ While seven NOX homologues have been characterized (NOX 1‐5 and Duox 1‐2), the Egea laboratory elegantly illustrated the importance of NOX4 utilizing a compound mutant *Fbn1^C1039G/+^‐Nox4* ^−^ *^/^* ^−^ mouse model, demonstrating reduced aneurysm size and decreased elastin breakdown compared to *Fbn1^C1039G/+^* mice.^28^, ^52^ Herein, we report that AngII regionally increases MFS SMC ROS production in AS‐derived SMC, but not in DES aorta‐derived SMC, in vitro. Previous studies similarly report that AngII can induce ROS in several cell types, including cardiomyocytes, cardiac fibroblasts and vascular SMC.^53^, ^54^, ^55^, ^56^ Because TGF‐β blockade reduced AngII‐dependent MFS AS SMC ROS production, we postulate that TGF‐β can increase oxidative stress directly, as well as indirectly via AngII. Kim et al analogously described that AngII increases SMC ROS production via ERK1/2 stimulation concomitantly while ROS induce ERK1/2 phosphorylation, thereby intensifying each other.^57^ Finally, we cannot rule out that AngII participates in aneurysm formation through an alternative mechanism, for example direct stimulation of MMP activation.^58^, ^59^ It is intriguing that the protective clinical benefit of losartan noted in several MFS clinical trials may be secondary to blockade of AngII‐stimulated ROS production.^60^, ^61^, ^62^, ^63^, ^64^, ^65^, ^66^, ^67^, ^68^, ^69^, ^70^

## CONFLICT OF INTEREST

None.

## AUTHOR CONTRIBUTION

Fabian Emrich and Kiril Penov contributed equally. Both authors performed the research, designed the study, analysed the data and wrote the paper. Mamoru Arakawa, Nathan Dhablania, Grayson Burdon, Albert J. Pedroza, Tiffany K. Koyano, Young M. Kim, Uwe Raaz and Andrew J. Connolly performed the research and analysed the data. Cristiana Iosef analysed the data and wrote the paper. Michael P. Fischbein designed the study, analysed the data and wrote the paper.

## Data Availability

Data are available upon request.
